# Influence of Ultrasound-Assisted and Supercritical CO_2_ Extraction on Phytochemical Profiles with Antimicrobial and Antioxidant Functionality from Olive Leaves and Olive Pomace

**DOI:** 10.3390/molecules31071186

**Published:** 2026-04-02

**Authors:** Yesuneh Gizaw, María José Benito, María de los Ángeles Rivas, Iris Gudiño, María de Guía Córdoba, Rocío Casquete

**Affiliations:** 1 Nutrición y Bromatología, Escuela de Ingenierías Agrarias, Universidad de Extremadura, Avd. Adolfo Suárez s/n, 06007 Badajoz, Spain; ygizawch@alumnos.unex.es (Y.G.); mrivasm@unex.es (M.d.l.Á.R.); igudino@unex.es (I.G.); mdeguia@unex.es (M.d.G.C.); rociocp@unex.es (R.C.); 2 Instituto Universitario de Investigación en Recursos Agrarios (INURA), Universidad de Extremadura, Avd. de la Investigación, 06006 Badajoz, Spain

**Keywords:** olive by-products, supercritical fluid extraction, ultrasound-assisted extraction, bioactive compounds, functional activities

## Abstract

This study evaluated olive leaves from three cultivars (Hojiblanca, Picual, and Arbequina) and olive pomace as complementary sources of bioactive compounds, comparing ultrasound-assisted extraction using organic solvents (UAE) with supercritical CO_2_ extraction (SFE). The aim was to determine how the plant matrix and extraction method influence phytochemical composition and functional properties, including antioxidant and antimicrobial activity. The results showed that both factors strongly affected extract composition and bioactivity. UAE favored the recovery of phenolic compounds associated with antioxidant activity, particularly in leaf extracts, while SFE promoted a distinct compositional profile enriched in flavonoids and lipophilic constituents, especially in olive pomace. Multivariate analysis confirmed a clear differentiation between matrices and extraction methods. Leaf extracts from Picual and Arbequina were mainly associated with phenolic compounds linked to antioxidant activity, including luteolin, ethyl vanillin, tyrosol, and isorhamnetin-3-O-glucoside. In contrast, olive pomace extracts were more strongly associated with flavonoids and lipophilic metabolites, such as triterpenes (oleanolic, maslinic, and ursolic acids) and lipid derivatives (oleic acid and lauric isopropanolamide). These compositional differences were reflected in biological activity: UAE extracts showed higher antioxidant activity, whereas SFE extracts, enriched in lipophilic and triterpenic compounds, exhibited stronger antimicrobial effects against *Pseudomonas savastanoi* and *Hanseniaspora* sp. Overall, these findings demonstrate that extraction-driven selectivity enables the production of olive-derived extracts with targeted functionalities, with UAE favoring antioxidant-oriented extracts and SFE promoting extracts enriched in lipophilic compounds with antimicrobial potential, particularly from olive pomace.

## 1. Introduction

Regular consumption of olive oil, within the context of a healthy diet, is associated with cardioprotective effects and a lower incidence of various chronic diseases. These benefits are primarily attributed to its fatty acid profile and the presence of minor bioactive constituents [1,2,3]. Consequently, in a context of high demand, olive oil production constitutes a strategic domain for the agri-food sector and international trade. In the 2024/25 season, global production was estimated at 3.572 million tons (Mt), of which the European Union contributed approximately 2.150 Mt and Spain around 1.430 Mt, confirming Spain as the leading European producer and one of the most relevant suppliers worldwide [4]. According to statistics from the Food and Agriculture Organization of the United Nations (FAO), the global harvested olive grove area is approximately 10.6 million ha [5], while official national statistics indicate that Spain has approximately 2.83 million ha of olive groves [6]. Overall, these figures underscore Spain’s central role as a key country and an international benchmark in olive oil production.

High production generates large amounts of by products that require adequate management. Their organic nature and frequent high moisture make them bulky. They degrade rapidly and can cause odors, while increasing storage, transport, and handling costs. These constraints hinder efficient management and large scale valorization [7,8]. Main residue streams include olive mill solid waste, olive mill wastewater, olive stones, and olive leaves removed during cleaning and handling [9,10]. Olive pomace is often the dominant fraction, with indicative generation around 0.8 kg per 0.2 kg of olive oil, depending on process configuration [11]. Although these residues are widely described as renewable feedstocks for recovery and reuse [12], implementation remains uneven and many by products are still routed to low value uses.

Among these matrices, olive leaves and olive pomace are particularly attractive due to their rich and heterogeneous composition [13]. They contain structural carbohydrates, proteins, minerals, residual lipids, and diverse minor bioactives that vary with cultivar, agronomy, harvest time, and processing conditions [14,15]. Phenolic compounds are especially abundant and extensively studied [16]. Olive leaves typically show high phenolic contents, dominated by secoiridoids such as oleuropein and derivatives, and phenolic alcohols including hydroxytyrosol and tyrosol [17]. Their phenolic profile is strongly affected by cultivar, agronomic practices, and harvest season [18,19,20]. Olive pomace retains relevant phenolics after oil extraction and also contains a significant terpenoid fraction [21,22]. This fraction is dominated by pentacyclic triterpenes, mainly oleanolic and maslinic acids, consistently detected in olive pomace, residual olive skin, and olive leaves [23,24,25]. The coexistence of phenolics and terpenoids supports integrated valorization strategies [8,26].

These olive-derived matrices have been extensively investigated for functional applications, with particular attention to their antioxidant and antimicrobial activities. Olive leaf extracts exhibit high antioxidant performance in food systems and can retard oxidative deterioration, justifying their use as natural preservatives [27]. In processed foods, olive leaf extract has also been applied to reduce lipid oxidation in baked snacks, highlighting its potential in formulations susceptible to rancidity [28]. Aqueous extracts derived from olive residues have demonstrated substantial antioxidant capacity, justifying their valorization as sources of protective compounds [29]. Beyond oxidative stability, antimicrobial activity has been described against food-relevant microorganisms, including Gram-positive and Gram-negative bacteria. Aqueous extracts derived from olive by-products have shown inhibitory effects and synergistic antimicrobial activity, with results consistent with alterations in cell integrity and effects related to oxidative stress in bacterial cells [30]. Specific components of olive pomace may contribute to the antimicrobial effects. Maslinic acid and oleanolic acid have been isolated from olive pomace and have demonstrated antibacterial activity in vitro [25]. Furthermore, studies on oleanolic acid and its derivatives indicate antibacterial activity in a variety of experimental systems [31].

In this study, *Pseudomonas savastanoi* and *Hanseniaspora* sp. were selected for antimicrobial testing because both are directly implicated in the physical and microbiological deterioration of olive leaves and fruits, with measurable consequences for technological and commercial quality. *P. savastanoi* can enter through wounds and colonize aerial olive organs, promoting tissue damage that weakens plant structures and increases nutrient leakage, thereby facilitating the proliferation of secondary microbiota [32]. Its detection in asymptomatic olive material further supports its relevance as a realistic bacterial target in preventive control strategies [33]. Conversely, *Hanseniaspora* sp. are part of the olive-associated mycobiota and have been reported among dominant fungal genera in fermented table olives [34]. However, uncontrolled yeast growth during processing and storage has been associated with undesirable metabolite production and quality defects such as off-flavors/off-odors, gas pocket formation and brine clouding [35].

Bioactivity depends largely on the extraction strategy, as processing conditions affect yield, chemical profile, and functional performance [36]. Therefore, extraction technology is crucial for valorization. Conventional solid–liquid extraction with organic solvents, typically hydroalcoholic mixtures, remains a benchmark for phenolic recovery and method comparison. Castillo-Correa et al. [37] reported, through a systematic review and quantitative synthesis of olive leaf extracts, that the extraction method, solvent system, and temperature significantly influence total phenolic recovery and associated antioxidant capacity. More recently, intensified and more environmentally friendly extraction technologies have been investigated to improve efficiency and fine-tune selectivity toward specific phytochemical fractions. Martín-García et al. [38] studies have shown that ultrasound-assisted extraction can improve the recovery of phenolic compounds from olive leaves while allowing for the evaluation of antioxidant and antimicrobial activities in the resulting extracts. Supercritical CO_2_ extraction represents another environmentally preferable alternative with adjustable selectivity, particularly suitable for more lipophilic constituents. Kyriakoudi et al. [39] demonstrated that supercritical CO_2_ extraction applied to olive leaves at 30 MPa, with temperature modulation between 35 and 90 °C and the use of ethanol and water as cosolvents, produced a value-added extract containing quantifiable bioactive compounds such as oleuropein, hydroxytyrosol, tyrosol, and α-tocopherol, with demonstrable functional activity.

Therefore, this study evaluated olive leaves from three different varieties and olive pomace as complementary sources of bioactive compounds and compared ultrasound-assisted extraction using organic solvents (UAE) with supercritical CO_2_ extraction (SFE). For each matrix and method, the total phenolic content, total flavonoid content, and individual compound profile were determined, along with antioxidant and antimicrobial activity against *Hanseniaspora* sp. and *Pseudomonas savastanoi*, to define the functional potential of the extracts and their possible use as natural bioactive ingredients.

## 2. Results and Discussion

### 2.1. Composition of the Extracts

As shown in Table 1, total phenolic content (TPC) was strongly influenced by both the extraction technique and the type of matrix analyzed. For olive leaves, ultrasound-assisted extraction yielded high TPC values ranging from 6753.26 to 7409.71 mg GAE/100 g for Picual, Hojiblanca, and Arbequina cultivars, with no significant differences among cultivars within this method (*p* > 0.05). In contrast, supercritical fluid extraction produced significantly lower TPC values in the same leaf samples (848.29–1763.46 mg GAE/100 g), indicating a reduced efficiency for recovering total phenolics from foliar material. A different pattern was observed for olive pomace, where supercritical fluid extraction yielded slightly higher TPC values (7659.97 mg GAE/100 g) than ultrasound-assisted extraction (7200.28 mg GAE/100 g), although these differences were not statistically significant (*p* > 0.05).

The results obtained are consistent with the literature. Under optimized ultrasound-assisted extraction of olive leaves, Uysal et al. [40] reported a total phenolic content of 8905 mg GAE/100 g; this value is of the same order of magnitude as that observed in our olive leaf extracts, although it is higher, which is expected under optimized extraction settings and considering the strong influence of the polar methanol/water system used in UAE on phenolic recovery. For olive pomace, Martínez-Patiño et al. [41] identified optimal ultrasound-assisted extraction conditions that yielded values of 5750 mg GAE/100 g of olive pomace; this value is consistent with our pomace results but lower, probably due to differences in pomace type, solvent composition, and extraction intensity, particularly the balance between polar and less polar fractions recovered in the two-step UAE procedure. For supercritical extraction of olive leaves, Kyriakoudi et al. [39] demonstrated a strong co-solvent effect, reporting total phenolic contents of up to 30,000 mg GAE/100 g when ethanol was used as a co-solvent at 30 MPa and 90 °C. This highlights that, in contrast to UAE, the efficiency of SFE for phenolic recovery is highly dependent on the introduction of polar modifiers to increase solvent polarity. Therefore, the lower phenolic recovery observed in our leaf extracts under supercritical conditions can be explained by the absence of a strong co-solvent, reinforcing the key role of solvent polarity and co-solvent selection in tuning extraction performance.

The behavior observed between ultrasound-assisted extraction (UAE) and supercritical CO_2_ extraction (SFE) can be explained not only by the different physicochemical principles governing these techniques, but also by the nature and polarity of the solvents involved. In the case of UAE, a methanol/water (80:20, *v*/*v*) mixture was used, providing a highly polar extraction medium that strongly favors the solubilization and recovery of polar phenolic compounds. In addition, the subsequent dichloromethane step allows the partial recovery of less polar constituents, resulting in a broader extraction spectrum. Ultrasound further intensifies mass transfer through cavitation, improving solvent penetration and facilitating the release of intracellular compounds, which is particularly relevant for polar phenolics in plant tissues such as olive leaves [42].

Conversely, supercritical CO_2_ is intrinsically non-polar, showing higher affinity for lipophilic or moderate polar constituents; therefore, it can be advantageous for complex matrices such as olive pomace that contain residual oils and structurally diverse phytochemicals, including polyphenols and other lipophilic fractions [43,44]. The limited efficiency observed in leaf samples can be attributed to the low solubility of highly polar phenols in neat CO_2_ and the strong dependence of phenolic recovery on the use and type of polar modifiers, as demonstrated for olive leaves under supercritical conditions [39]. In addition, supercritical processing has been applied to multiple olive residues, highlighting that optimal supercritical conditions and solvent tuning are residue dependent [45].

Therefore, the differences observed between UAE and SFE should not be interpreted exclusively as a consequence of the extraction technique itself, but rather as the result of the combined effect of extraction mechanism and solvent polarity. In this context, UAE provides a more polar environment that promotes phenolic recovery, whereas SFE without co-solvent favors the extraction of less polar compounds. Overall, these findings underline the importance of considering both solvent characteristics and extraction technology when selecting an appropriate strategy for the valorization of olive industry by-products.

Table 2 reports the flavonoid content, expressed as mg quercetin equivalents (QE) per 100 g dry sample, measured in olive leaves from the studied cultivars and in olive pomace after ultrasound-assisted extraction and supercritical fluid extraction.

As shown in Table 2, total flavonoid content (TFC), like TPC, was significantly affected by both the extraction technique and the matrix. For all olive leaf samples, SFE yielded significantly higher TFC than UAE (*p* < 0.05), increasing from 141.47 to 181.71 mg QE/100 g to 318.65–806.94 mg QE/100 g. With UAE, differences between leaf cultivars were limited, whereas with SFE, a clear cultivar effect emerged, with Arbequina exhibiting the highest leaf TFC (806.94 mg QE/100 g). Olive pomace followed the same method-dependent trend, increasing from 237.27 mg QE/100 g (UAE) to 956.38 mg QE/100 g (SFE), and exhibited the highest values within each method, indicating that pomace is a matrix more enriched with flavonoids than leaves under the tested conditions.

These findings are consistent with previous evidence that olive-derived matrices are relevant sources of flavonoids and that extraction conditions can substantially influence their recovery [46]. In our dataset, olive pomace consistently showed a higher total flavonoid content than leaves, supporting the idea that flavonoids are largely retained in the solid residue during olive oil production rather than being transferred to the final oil [47]. Furthermore, the magnitude of our pomace values is consistent with previous reports. For olive pomace, Niknam et al. [48] reported a total flavonoid content of 2.48 mg QE per gram in ultrasound-treated pomace extracts, corresponding to 248 mg QE per 100 g, which closely matches the value observed for pomace subjected to ultrasound-assisted extraction in Table 2.

The higher TFC obtained by supercritical extraction in the different matrices can be attributed to differences in selectivity between the methods. Carbon dioxide-based supercritical processes are highly condition-dependent and can favor the recovery of specific flavonoid fractions under appropriate operating conditions, as demonstrated in olive leaf extracts [39]. Furthermore, comparative evidence in other plant matrices indicates that supercritical CO_2_ extraction can yield a higher total flavonoid content than ultrasound-assisted extraction, supporting the results obtained in this study [49].

The identification results and relative abundances of bioactive compounds in olive by-product extracts, as determined by HPLC–ESI–QTOF analysis, are reported in Table 3 and Table 4. Table 3 provides a systematic classification of the identified constituents and includes, for each compound, the corresponding retention time (RT) and diagnostic MSn fragment ions. Table 4 presents the relative abundance of the detected compounds.

The identification (Table 3) and relative abundance (Table 4) of bioactive compounds by HPLC-ESI-QTOF in olive by-products reveal several interesting patterns; the compounds reported represent the predominant detected constituents, whereas additional minor metabolites were also observed but are not included here.

Leaf samples are mainly characterized by phenolic and flavonoid markers (tyrosol-related signals and luteolin-type compounds), which agree with recent high-resolution profiling studies reporting oleuropein-related secoiridoids together with simple phenols and flavonoids as prominent constituents of olive leaves [55]. In addition, cultivar-driven variability in the leaf phenolic fingerprint is consistent with reports showing that olive-leaf phenolic profiles can differ measurably among cultivars and sites, including variation in flavonoid-type features [19]. In pomace, the appearance of triterpenic acids (oleanolic/maslinic/ursolic acids) alongside phenolic constituents such as verbascoside and hydroxytyrosol is in line with tissue-specific distributions described for olive by-products, where pomace is recognized as an important reservoir of these phenolics and triterpenoids [56]. Finally, the co-occurrence of lipophilic features in pomace matches compositional characterizations of olive pomace that emphasize its residual lipid fraction together with phenolic/secoiridoid components [57].

In Table 4, the chromatographic fingerprints of UAE and SFE show clear differences in selectivity, but these should be interpreted as relative patterns, as the data are expressed in arbitrary peak area units. In leaf samples, UAE is characterized primarily by signals assigned to more polar phenolic compounds and flavonoids, which is consistent with the suitability of ultrasonic extraction for recovering phenolic components from olive leaves with organic solvents [38].

In contrast, the most distinctive change driven by SFE is observed in olive pomace, where the profile is relatively enriched in lipophilic fractions, including fatty acid-like signals and triterpenic acids, reflecting the affinity of supercritical carbon dioxide for less polar constituents and the influence of residual oil domains in the pomace matrix [58]. The occurrence of triterpenic acids primarily in pomace extracts is consistent with reports showing that supercritical approaches can efficiently recover triterpenes and related nonpolar fractions from olive-derived residues, particularly when the target matrix contains lipophilic domains that enhance solubilization and partitioning [59,60]. Overall, these qualitative signatures indicate that UAE favors polar phenolic-type characteristics, while SFE in pomace selectively enriches more lipophilic families, underscoring the importance of tailoring the extraction strategy to the matrix composition and the target compound class.

### 2.2. Antioxidant Activity

The results of the antioxidant activity of the studied olive by-products, quantified through the 2,2-diphenyl-1-picrylhydrazyl (DPPH) radical-scavenging assay and the 2,2′-azinobis-(3-ethylbenzothiazoline)-6-sulfonic acid (ABTS) radical-scavenging assay, are presented in mg of Trolox per 100 g of extract in Table 5.

As shown in Table 5, antioxidant activity was highly dependent on the extraction method and matrix. Ultrasonic extraction yielded significantly higher DPPH and ABTS values than supercritical fluid extraction in all samples, with significant differences within each matrix (*p* < 0.05). For DPPH, UAE produced high mean activities in the range of 6199.34 to 7158.31 mg Trolox/100 g, whereas SFE values were much lower, ranging from 91.87 to 626.00 mg Trolox/100 g. For ABTS, UAE values ranged from 3845.96 to 5562.00 mg Trolox/100 g, while SFE values were again markedly reduced, spanning 91.01 to 592.26 mg Trolox/100 g, with the lowest mean activity observed for olive pomace under SFE.

This pattern is consistent with the ability of ultrasound to intensify mass transfer by cavitation. It enhances solvent penetration and promotes the release of intracellular antioxidants in the extract phase [42]. The scientific literature supports the magnitude of these responses under intensified extraction. Martínez-Patiño et al. [61] reported mean DPPH values of 42.5 mg Trolox per g and ABTS values of 95.9 mg Trolox per g for olive leaf-derived material processed by ultrasound under optimized conditions. For exhausted olive pomace extracts, Gómez-Cruz et al. [62] reported DPPH values of 25.8 to 49.2 mg Trolox per g and ABTS values of 93.4 to 142.9 mg Trolox per g, confirming that phenolic-rich pomace extracts can exhibit very high antiradical capacity when extraction is effective. Lower values have also been observed when extraction conditions are less favorable for phenolic release, as summarized in the literature for pomace processes [63].

Chemical signatures help explain the antioxidant capacity results. UAE profiles are dominated by more polar phenolics and flavonoid-related signals. These include hydroxytyrosol and tyrosol like features and flavonoids such as luteolin. Luteolin also exhibits marked scavenging capacity in common antioxidant assay systems, supporting its contribution when its signal is prominent [64]. In olive pomace, the presence of verbascoside as a major UAE-associated feature provides an additional mechanistic link, as verbascoside exhibits strong free radical-scavenging capacity [65]. Conversely, the much lower antioxidant values after SFE are consistent with a shift towards more lipophilic fractions and a reduced representation of the more reactive polar phenolic scavengers. It is known that supercritical CO_2_ extraction of olive pomace recovers lipophilic phytocompounds and the balance between these classes depends on the conditions, which can result in extracts that are not dominated by polar radical scavengers [44]. In addition, the extraction temperature applied (80 °C) may contribute to the partial degradation of thermolabile phenolic compounds, which could further reduce the overall antioxidant capacity of the SFE extracts.

### 2.3. Antimicrobial Activity

Table 6 and Table 7 summarize the antimicrobial performance of olive-derived extracts obtained by SFE and UAE against two relevant plant-associated microorganisms that cause spoilage: *Pseudomonas savastanoi* (antibacterial assay) and *Hanseniaspora* sp. (antifungal assay). In both assays, activity was evaluated at three extract concentrations (70, 140, and 280 ppm) and for two matrices (olive leaves of the Hojiblanca, Picual, and Arbequina varieties, and olive pomace), allowing for a direct comparison of the extraction strategy, the dose–response relationship, and the effects of the matrix.

In Table 6, the inhibition of *Pseudomonas savastanoi* was clearly influenced by extract concentration, plant matrix, and extraction method. At the highest tested concentration (280 ppm), SFE extracts produced almost complete inhibition in all matrices (100%), whereas UAE extracts showed lower and more variable inhibition, ranging from 76% in Picual leaves to 100% in Arbequina leaves and olive pomace (*p* < 0.05). At 140 ppm, SFE extracts maintained very high inhibition (96–100%) across all matrices, while UAE extracts decreased markedly, showing values between 58% and 97%, depending on the matrix. At the lowest concentration (70 ppm), the differences between extraction methods became more pronounced: SFE extracts still showed moderate to high inhibition (59–93%), whereas UAE extracts dropped substantially (18–75%), particularly in Picual leaves. These results indicate that SFE extracts maintain antibacterial efficacy even at lower concentrations, while UAE extracts show a clearer concentration-dependent response. This pattern is consistent with the idea that olive phenolics can suppress *P. savastanoi* not only via direct growth effects but also by interfering with virulence regulation, including quorum sensing and type three secretion, depending on extract composition and dose [66].

The stronger activity of SFE at the lowest tested concentration is plausible given the qualitative enrichment in less polar constituents typically recovered by supercritical carbon dioxide from olive residues, including triterpenic acids and fatty acid-like fractions. Pentacyclic triterpenes such as oleanolic and ursolic acids have documented antibacterial activity and proposed mechanisms involving envelope stress and interference with essential bacterial processes, which can enhance effectiveness against challenging targets [67]. In addition, oleanolic-acid-derived triterpenic compounds have been tested for antibacterial activity including against *Pseudomonas* species, supporting the relevance of this compound class for anti-*Pseudomonas* effects when present in an extract [68]. By contrast, UAE extracts are expected to be richer in polar phenolics and flavonoids, which can show antibacterial activity against *P. savastanoi* but often in a concentration-dependent manner and with possible synergy among minor components [69]. Recent work specifically addressing olive knot pathogens also supports the antibacterial potential of olive-derived phenolic systems, including hydroxytyrosol and phenolic-rich olive mill streams, while emphasizing that efficacy varies with phenolic profile and applied concentration [70].

In Table 7, antifungal activity against *Hanseniaspora* sp. was strongly influenced by extraction method, concentration, and matrix. SFE extracts showed consistently high antifungal activity across all matrices, even at the lowest concentration tested. At 70 ppm, inhibition remained very high, reaching 97–97% for Hojiblanca and olive pomace, 64% for Picual, and 78% for Arbequina. At 140 ppm, SFE extracts maintained strong inhibition (75–99% depending on the matrix), while at 280 ppm the inhibition was almost complete (97–100%) for all matrices.

In contrast, UAE extracts displayed markedly lower antifungal activity and a clear concentration-dependent response. At 70 ppm, inhibition was null or negligible (0–8%), and at 140 ppm it remained very limited (0–29% depending on the matrix). A noticeable increase was only observed at 280 ppm, where inhibition ranged from 40% to 65%, still considerably lower than the activity observed for SFE extracts.

This pattern is consistent with previous studies indicating that olive-derived extracts can inhibit yeast growth, although their effectiveness strongly depends on extraction conditions, chemical composition, and applied concentration. For instance, Korukluoglu et al. [71] reported antifungal effects of olive leaf extracts against yeast species and highlighted that extraction method and solvent polarity significantly influence antifungal efficacy, supporting the strong differences observed here between SFE and UAE extracts.

The stronger SFE effect at the lowest dose is plausible if the extracts are relatively enriched in lipophilic antifungal constituents. Fatty acids and their derivatives are widely reported to inhibit fungi and yeasts, mainly through membrane-related effects, which supports a potential contribution when fatty acid-like fractions are prominent [72]. Pentacyclic triterpenes can also display direct antifungal activity; oleanolic acid has been reported to inhibit *Candida albicans* isolates with minimum inhibitory concentration (MIC) values of 32–64 µg/mL, indicating that triterpene-enriched fractions may contribute to strong inhibition at low extract concentration [73]. For more polar systems, aqueous olive leaf extracts have shown antifungal effects against *C. albicans* at mg/mL-range MICs, consistent with the need for higher concentrations to achieve marked inhibition when polar phenolics dominate [74]. In addition, luteolin has documented activity against *C. albicans* in vitro, supporting the potential contribution of flavonoid-type constituents when sufficiently concentrated in the extract [75].

### 2.4. Multivariate Analysis

**Figure 1:**
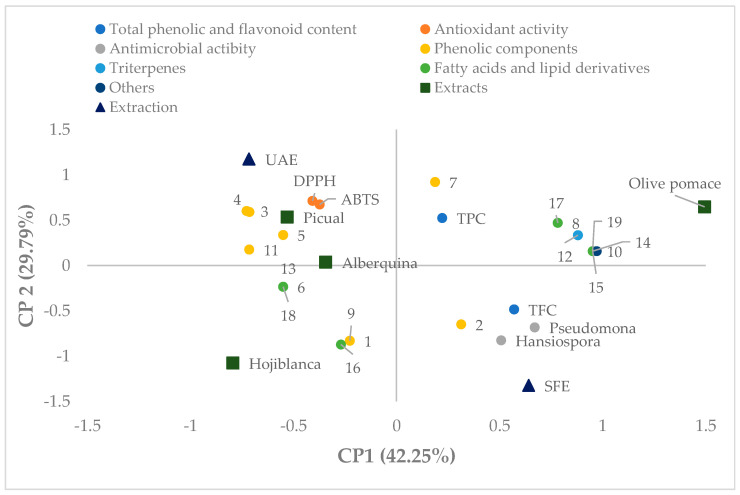
Projection on the plane defined by principal components 1 and 2 of the principal component analysis (PCA) of the analytical parameters and identified compounds (total phenols—TPC, total flavonoids—TFC, antioxidant activity—DPPH and ABTS; antimicrobial activity against *Pseudomonas* and *Hanseniaspora*, and compounds 1–20 (Table 3)).

The principal component analysis (Figure 1) indicates that the first two principal components explain 72.04% of the total variability, with PC1 accounting for 42.25% and PC2 for 29.79%.

The positive axis of principal component 1 (PC1) is mainly associated with antimicrobial activity against *Pseudomonas* and *Hanseniaspora*, as well as with several lipidic and triterpenic compounds, including oleanolic acid (12), maslinic acid (14), ursolic acid (15), verbascoside (8), lauric isopropanolamide (17), and oleic acid (19). These parameters and compounds are clustered on the right-hand side of the plot, close to the olive pomace sample, indicating a strong relationship between this matrix and the content of lipidic and triterpenic compounds as well as antimicrobial activity.

In contrast, the negative side of PC1 is related to phenolic compounds such as luteolin (3), ethyl vanillin (4), tyrosol (5), and isorhamnetin-3-O-glucoside (11). These variables are located close to the Picual samples, suggesting that these matrices are more strongly associated with phenolic compounds responsible for antioxidant activity.

The second principal component (PC2) mainly differentiates the effect of the extraction method. In the upper part of the plot, parameters related to antioxidant activity and extraction efficiency (UAE) are located, together with some phenolic compounds such as 2,4,6-trimethylbenzoic acid (7). Conversely, the negative axis of PC2 is associated with the supercritical fluid extraction method (SFE) and with lipidic compounds and fatty acids, such as 12-HETE (16), as well as some phenolic derivatives such as Q-3-O-diglucoside (9).

Furthermore, a clear separation between the different plant matrices evaluated can be observed. Hojiblanca is located in the lower-left quadrant, mainly associated with certain fatty acids and specific phenolic compounds, whereas Picual and Arbequina appear closer to the antioxidant activity parameters. In contrast, olive pomace clearly differs from the other samples, showing a stronger association with triterpenic and lipidic compounds as well as antimicrobial activity.

Therefore, the principal component analysis confirms the differences among the studied matrices and extraction methods, showing that extracts obtained by supercritical fluids are more closely related to lipidic and triterpenic compounds, whereas ultrasound-assisted extracts are mainly associated with phenolic compounds and antioxidant activity, thus corroborating the analytical results previously obtained in this study.

## 3. Materials and Methods

### 3.1. Plant Material

Olive oil by-products were obtained from an olive oil processing plant located in Extremadura, Spain. Olive leaves from three cultivars (*Olea europaea* L. cvs. Hojiblanca, Picual, and Arbequina) were collected during routine olive cleaning prior to oil extraction, while olive pomace was collected immediately after oil extraction. Despite the intrinsic differences between these matrices, both sample types were subjected to the same pre-treatment procedure in order to ensure comparability between extraction methods. Samples were transported under refrigeration and, upon arrival, were dried in a forced-air oven at 45 °C (J.P. Selecta Digitronic, Abrera, Spain) to constant weight, ground to a fine powder, sieved, and stored at −20 °C until extraction.

### 3.2. Extraction of Bioactive Compounds

Extractions were performed in triplicate using two methods: ultrasound-assisted solvent extraction and supercritical CO_2_ extraction.

#### 3.2.1. Ultrasound-Assisted Solvent Extraction

Solvent extraction was performed using a two-step protocol adapted from Zhang et al. [76] to recover phenolics and triterpenoids. Due to this protocol, 0.5 g of dried sample was mixed with 10 mL of methanol/water (80:20, *v*/*v*) and sonicated at 40 kHz for 60 min (J. P. Selecta, Abrera, Spain), with temperature controlled, 20–35 °C, using an ice bath. The mixture was centrifuged at 2.000× *g* for 10 min, and the supernatant was collected. The remaining solid residue was subsequently re-extracted with 5 mL dichloromethane to enhance recovery of pentacyclic triterpenoids, followed by sonication at 40 kHz for 60 min and centrifugation at 2.000× *g* for 10 min. Organic solvents were removed under reduced pressure at 37 °C using a rotary evaporator (Büchi R-20, Büchi Labortechnik AG, Flawil, Switzerland), and the resulting extracts were stored at −20 °C until analysis.

#### 3.2.2. Supercritical CO_2_ Extraction

Supercritical fluid extraction was performed using a Spe-ed SFE system (Helix Applied Separations, Allentown, PA, USA) operating in dynamic mode. Forty grams of dried sample were loaded into a 100 mL stainless-steel extraction vessel, forming a fixed bed to ensure uniform solvent percolation. The CO_2_ flow rate was set at 2 L min^−1^ throughout the extraction. Extractions were carried out at 350 bar and 80 °C for 50 min. These operating conditions were selected on the basis of preliminary trials aimed at maximizing extract recovery and bioactive yield while maintaining process stability and minimizing the risk of thermal degradation. The resulting extracts were collected in glass vials and stored at −80 °C until analysis.

### 3.3. Characterization of the Extracts

#### 3.3.1. Determination of Total Phenolic Compounds Content

Total phenolic content was determined using the Folin–Ciocalteu spectrophotometric assay following Casquete et al. [77]. An aliquot of extract (0.5 mL) was transferred to a 25 mL volumetric flask and diluted with Milli-Q water (10 mL). Folin–Ciocalteu reagent (1.0 mL) was then added, followed by saturated sodium carbonate solution (2.0 mL), and the mixture was brought to volume with Milli-Q water and mixed thoroughly. After incubation for 60 min at room temperature in the dark, absorbance was measured at 760 nm. Quantification was performed using a gallic acid calibration curve, and results were expressed as mg gallic acid equivalents per 100 g dry sample (mg GAE/100 g).

#### 3.3.2. Determination of Total Flavonoid Content

Total flavonoid content was determined by the aluminium chloride colorimetric assay following Shraim et al. [78]. A 1.0 mL aliquot of appropriately diluted extract was combined with 3.0 mL methanol, then 0.2 mL AlCl_3_ solution 10% *w*/*v* and 0.2 mL potassium acetate 1 M were added, followed by 5.6 mL distilled water to obtain the final reaction mixture. After incubation for 30 min at room temperature in the dark, absorbance was recorded at 415 nm. Quantification was carried out using a quercetin calibration curve, and results were expressed as mg quercetin equivalents per 100 g of sample dry sample (mg QE/100 g).

#### 3.3.3. Identification of Bioactive Compounds

To characterize the phytochemical composition of each extract, samples were diluted in LC–MS grade methanol to a final concentration of 100 ppm and passed through a 0.45 μm syringe filter prior to analysis. The resulting solutions were analyzed using an HPLC system coupled to a quadrupole time-of-flight mass spectrometer (HPLC–QTOF, Agilent G6530; Agilent Technologies, Palo Alto, CA, USA). Chromatographic separation was performed on a C18 column 4.6 mm × 150 mm with 4.8 μm particle size. Detection and tentative identification of bioactive constituents were carried out by Q-TOF tandem mass spectrometry using an electrospray ionization source operating in negative ion mode. The gas flow was 11 mL/min at 280 °C (nebulizer 35 psi). Gradient elution was carried out with a mixture of hydrocyanic acid/water (5:95, *v*/*v*) as solvent A and hydrocyanic acid/water/formic acid (95:4.9:0.1 *v*/*v*/*v*) as solvent B, with a flow rate of 0.350 mL/min. The solvent gradient started with solvent B/solvent A (5:95, *v*/*v*), reaching 90:10 (*v*/*v*) at 15 and 20 min, and returning to the initial conditions for the last 10 min. Putative assignments were established by matching accurate-mass and MS/MS information against the MassBank database using Qualitative Analysis software, version B.07.00 [50]. Mass spectral data are reported as *m*/*z* values corresponding to deprotonated molecular ions [M−H]^−^ acquired in negative ESI mode, including both nominal and exact masses depending on the compound and identification criteria.

### 3.4. Antioxidant Capacity of the Extracts

Antioxidant capacity of the extracts was assessed using two complementary radical-scavenging assays, DPPH and ABTS. For the DPPH assay, an aliquot of extract (50 µL) was added to DPPH working solution (2.95 mL) in a cuvette, mixed, and allowed to react for 30 min at room temperature in the dark; absorbance was then recorded at 515 nm, as adapted from Kondo et al. [79]. For the ABTS assay, the ABTS radical cation solution (1.00 mL) was combined with the extract (20 µL) in a cuvette, and absorbance was measured at 730 nm immediately and after 20 min of reaction, following the procedure described by Arnao et al. [80] with minor adjustments. Trolox was used for calibration, and results were expressed as mg Trolox per 100 g of extract.

### 3.5. Antimicrobial Activity of the Extracts

Antimicrobial activity was evaluated against a bacterial and a yeast strains implicated in the physical and microbiological deterioration of olive leaves and fruits. *Pseudomonas savastanoi* was obtained from the Spanish Type Culture Collection (CECT), while *Hanseniaspora* sp. came from the microbial collection of the School of Agricultural Engineering at the University of Extremadura. The bacteria were cultured in brain–heart infusion (BHI) broth at 37 °C for 24 h, and the yeast was cultured in yeast peptone-dextrose (YPD) broth at 25 °C for 72 h. Both cultures were standardized to 1 × 10^7^ CFU mL^−1^ and dispensed into sterile 96-well microplates containing serial concentrations of the extracts (70,140 and 280 ppm). The plates were incubated under the respective growth conditions, and microbial growth was monitored spectrophotometrically at 560 nm using a microplate reader (FLUOstar, BMG Labtech, Ortenberg, Germany). Antimicrobial activity was expressed as the percentage of growth inhibition relative to untreated controls.

### 3.6. Statistical Analysis

Statistical analysis of the data was performed using SPSS for Windows, version 21.0 (IBM Corp., Armonk, NY, USA). Descriptive statistics were determined. For parameters related to general bioactivity and chemical composition, differences within and between groups were assessed using one, two, and three-way analysis of variance (ANOVA), and averages were separated using Tukey’s Honestly Significant Difference test (*p* < 0.05). Principal component analysis (PCA) was conducted to explore multivariate relationships among extraction method, sample type, and the measured response variables.

## 4. Conclusions

The results clearly demonstrate that both the extraction method and the plant matrix strongly determine the chemical composition and functional properties of the obtained extracts. Ultrasound-assisted extraction (UAE) favored the recovery of phenolic compounds associated with antioxidant activity, particularly in Picual and Arbequina leaf samples, which were closely linked to metabolites such as luteolin, ethyl vanillin, tyrosol, and isorhamnetin-3-O-glucoside. In contrast, olive pomace extracts exhibited a distinct chemical profile, characterized by a stronger association with flavonoids and lipophilic compounds, including triterpenes (oleanolic, maslinic, and ursolic acids) and lipid derivatives such as oleic acid and lauric isopropanolamide. These compositional differences were reflected in their biological functionality, as extracts enriched in lipophilic and triterpenic compounds showed the strongest antimicrobial activity against *Pseudomonas savastanoi* and *Hanseniaspora* sp., while UAE extracts were mainly associated with phenolic compounds and antioxidant activity.

Overall, these results highlight that extraction-driven selectivity allows the production of olive-derived extracts with targeted functionalities, with UAE favoring antioxidant-rich extracts and SFE promoting extracts enriched in lipophilic compounds with antimicrobial potential, particularly from olive pomace.

## Figures and Tables

**Table 1:** Total phenolic content (TPC), expressed as mg gallic acid equivalents (GAE) per 100 g dry sample, obtained by ultrasound-assisted extraction and supercritical fluid extraction from olive leaves of the studied cultivars and from olive pomace.

TPC (mg GAE per 100 g Dry Sample)						
Extract	Ultrasound-Assisted Extraction			Supercritical Fluid Extraction		
	Mean		SD *	Mean		SD
Hojiblanca	6973.25	±	263.85 ^a^	848.29	±	152.42 ^b^_1_
Picual	6753.26	±	200.52 ^a^	1253.08	±	479.08 ^b^_1_
Arbequina	7409.71	±	451.20 ^a^	1763.46	±	419.07 ^b^_1_
Olive pomace	7200.28	±	372.23 ^a^	7659.97	±	844.77 ^a^_2_

* SD: standard deviation. Superscript (a, b): mean values with different letters indicate significant differences (*p* < 0.05) between extraction methods within the same sample. Subscript (1, 2): mean values with different numbers indicate significant differences (*p* < 0.05) among samples within the same extraction method.

**Table 2:** Total flavonoid content (TFC), expressed as mg quercetin equivalents (QE) per 100 g dry sample, obtained by ultrasound-assisted extraction and supercritical fluid extraction from olive leaves of the studied cultivars and from olive pomace.

TFC (mg QE per 100 g Dry Sample)						
Extract	Ultrasound-Assisted Extraction			Supercritical Fluid Extraction		
	Mean		SD *	Mean		SD
Hojiblanca	141.47	±	8.07 ^b^_1_	318.65	±	88.51 ^a^_1_
Picual	181.71	±	6.50 ^b^_1,2_	429.16	±	11.12 ^a^_2_
Arbequina	179.91	±	14.83 ^b^_1,2_	806.94	±	1.56 ^a^_3_
Olive pomace	237.27	±	70.55 ^b^_2_	956.38	±	48.87 ^a^_4_

* SD: standard deviation. Superscript (a, b): mean values with different letters indicate significant differences (*p* < 0.05) between extraction methods within the same sample. Subscript (1, 2, 3, 4): mean values with different numbers indicate significant differences (*p* < 0.05) among samples within the same extraction method.

**Table 3:** Relative abundance (peak area, arbitrary units) of compounds identified in olive by-product extracts obtained by ultrasound-assisted extraction (UAE) and supercritical fluid extraction (SFE) from olive leaves (Hojiblanca, Picual, Arbequina) and olive pomace analyzed by HPLC-ESI-QTOF.

Peak	Rt (min)	[M−H]^−^	MS/MS (*m*/*z*, Negative ESI Mode)	Compound Identified
Phenolic components				
1	3.27	**315**	108; 109	Protocatechuic acid glucoside ^a^
2	14.82	**153**	-	Hydroxytyrosol ^a^
3	14.86	**287**	135; 153	Luteolin ^b^
4	14.98	**165**	123; 151; 193	Ethyl vanillin ^c^
5	15.06	**137**	119; 138	Tyrosol ^c^
6	16.82	**271**	119; 153; 272	Naringenin ^d^
7	19.39	**119**	107; 135; 141; 437	2,4,6-Trimethylbenzoic acid ^a^
8	20.08	**623**	277; 624; 625	Verbascoside ^e^
9	20.40	**625**	258; 468; 626; 641	Q 3-O-diglucoside ^a^
10	23.06	**297**	298	3′,7-Dimethoxy-3-hydroxyflavone ^a^
11	25.76	**477**	133; 189; 201	Isorhamnetin-3-O-glucoside ^a^
Triterpenes				
12	17.389	**119**	-	Oleanolic acid ^a^
13	19.509	**511**	512	3-O-Acetyl-16alpha-hydroxydehydrotrametenolic acid ^a^
14	21.734	**471**	-	Maslinic acid ^a^
15	25.465	**455**	-	Ursolic acid ^a^
Fatty acids and lipid derivatives				
16	20.695	**203**	149; 161; 173; 175; 429	12-HETE ^a^
17	21.187	**256**	102; 257	Lauric isopropanolamide ^a^
18	22.859	**283**	102; 285; 595	Stearic acid ^a^
19	22.629	**281**	-	Oleic acid ^a^
Others				
20	14.304	**139**	-	Oleoside ^a^

Values correspond to the *m*/*z* of deprotonated molecular ions [M−H]^−^ acquired in negative electrospray ionization (ESI^−^) mode. Both nominal and exact mass values are reported, depending on the compound and data processing conditions. The most representative ion is highlighted in bold within the [M−H]^−^ column. Compound identification was supported by comparison with MassBank and literature data ^a^ MassBank [50]; ^b^ Olmo-García et al. [51]; ^c^ Kalagiouri et al. [52]; ^d^ Tamasi et al. [53]; ^e^ Benincasa et al. [54].

**Table 4:** Relative abundances in arbitrary area units of the compound profile obtained by ultrasound-assisted extraction (UAE) and supercritical fluid extraction (SFE) from olive leaves (Hojiblanca, Picual and Arbequina) and olive pomace.

	Hojiblanca		Picual		Arbequina		Olive Pomace	
	SFE	UAE	SFE	UAE	SFE	UAE	SFE	UAE
Phenolic components								
1	17.68	nd	nd	nd	nd	nd	nd	nd
2	* nd	nd	nd	nd	nd	nd	76.69	nd
3	nd	133.01	nd	164.60	nd	227.73	nd	22.90
4	nd	91.28	nd	136.19	nd	208.26	nd	12.71
5	nd	97.07	nd	41.82	nd	427.25	nd	nd
6	nd	16.45	nd	nd	nd	nd	nd	nd
7	nd	nd	nd	280.42	nd	319.91	nd	275.59
8	nd	nd	nd	nd	nd	nd	nd	206.93
9	288.15	nd	nd	nd	nd	nd	nd	nd
10	nd	nd	nd	nd	nd	nd	671.17	nd
11	nd	58.55	nd	34.59	nd	nd	nd	nd
Triterpenes								
12	nd	nd	nd	nd	nd	nd	13.39	15.13
13	nd	15.85	nd	nd	nd	nd	nd	nd
14	nd	nd	nd	nd	nd	nd	170.21	nd
15	nd	nd	nd	nd	nd	nd	107.07	nd
Fatty acids and lipid derivatives								
16	608.29	nd	188.21	nd	201.17	nd	nd	nd
17	nd	nd	nd	nd	nd	nd	nd	782.66
18	nd	671.82	nd	nd	nd	nd	nd	nd
19	nd	nd	nd	nd	nd	nd	nd	nd
Others								
20	nd	nd	nd	nd	nd	nd	89.76	nd

The numerical code corresponds to the compounds listed in Table 3. * nd—non detected.

**Table 5:** Antioxidant activity determined by DPPH and ABTS (mg Trolox per 100 g dry sample) assays for extracts obtained by ultrasound-assisted extraction (UAE) and supercritical fluid extraction (SFE) from olive leaves (Hojiblanca, Picual and Arbequina) and olive pomace.

Antioxidant Activity (mg Trolox per 100 g Dry Sample)												
Extract	DPPH						ABTS					
	UAE			SFE			UAE			SFE		
	Mean		SD *	Mean		SD	Mean		SD	Mean		SD
Hojiblanca	6199.34	±	125.19 ^a^	343.62	±	44.81 ^b^_2_	5132.99	±	249.82 ^a^	505.64	±	12.98 ^b^_1_
Picual	7055.04	±	575.38 ^a^	626.00	±	90.40 ^b^_3_	4628.27	±	35.69 ^a^	481.66	±	33.84 ^b^_1_
Arbequina	7158.31	±	1417.93 ^a^	304.52	±	21.13 ^b^_2_	3845.96	±	285.51 ^a^	592.26	±	22.31 ^b^_1_
Olive pomace	6863.24	±	1062.55 ^a^	91.87	±	2.86 ^b^_1_	5562.00	±	71.38 ^a^	91.01	±	46.25 ^b^_2_

* SD: standard deviation. Superscript (a, b): mean values with different letters indicate significant differences (*p* < 0.05) between extraction methods within the same sample. Subscript (1, 2, 3): mean values with different. numbers indicate significant differences (*p* < 0.05) among samples within the same extraction method.

**Table 6:** Antibacterial activity of extracts obtained by supercritical fluid extraction (SFE) and ultrasound-assisted extraction (UAE) against *Pseudomonas savastanoi* (growth inhibition. % vs. untreated control).

Extract	Hojiblanca						Picual						Arbequina						Olive Pomace					
	SFE			UAE			SFE			UAE			SFE			UAE			SFE			UAE		
	Mean		SD *	Mean		SD	Mean		SD	Mean		SD	Mean		SD	Mean		SD	Mean		SD	Mean		SD
Extract concentration																								
280 ppm	100	±	1.1 ^a^_1_	93	±	3.5 ^b^_1_	100	±	4.0 ^a^_1_	76	±	2.0 ^b^_1_	100	±	1.3 ^a^_1_	98	±	1.5 ^a^_1_	100	±	1.3 ^a^_1_	100	±	2.1 ^a^_1_
140 ppm	100	±	4.0 ^a^_1_	71	±	3.2 ^b^_2_	96	±	3.2 ^a^_1_	58	±	5.4 ^b^_2_	98	±	1.9 ^a^_1_	64	±	6.5 ^b^_2_	99	±	2.6 ^a^_1_	97	±	2.5 ^a^_2_
70 ppm	68	±	6.2 ^a^_2_	44	±	7.8 ^b^_3_	59	±	2.5 ^a^_2_	18	±	10.0 ^b^_3_	72	±	7.9 ^a^_2_	57	±	4.7 ^b^_3_	93	±	6.7 ^a^_1_	75	±	10.1 ^b^_3_

* SD: standard deviation. Superscript (a, b): mean values with different letters indicate significant differences (*p* < 0.05) between extraction methods within the same sample. Subscript (1, 2, 3): mean values with different numbers indicate significant differences (*p* < 0.05) among extract concentrations within the same extraction method.

**Table 7:** Antifungal activity of extracts obtained by supercritical fluid extraction (SFE) and ultrasound-assisted extraction (UAE) against *Hanseniaspora* sp. (growth inhibition. % vs. untreated control).

	Hojiblanca						Picual						Arbequina						Olive Pomace					
Extract	SFE			UAE			SFE			UAE			SFE			UAE			SFE			UAE		
	Mean		SD *	Mean		SD	Mean		SD	Mean		SD	Mean		SD	Mean		SD	Mean		SD	Mean		SD
*Concentration*																								
280 ppm	100	±	0.3 ^a^_1_	43	±	1.5 ^b^_1_	98	±	1.3 ^a^_1_	65	±	9.1 ^b^_1_	97	±	1.3 ^a^_1_	40	±	1.5 ^b^_1_	100	±	0.0 ^a^_1_	64	±	0.0 ^b^_1_
140 ppm	99	±	0.9 ^a^_1_	10	±	1.2 ^b^_2_	75	±	2.8 ^a^_2_	13	±	5.4 ^b^_2_	81	±	0.1 ^a^_2_	0	±	0.1 ^b^_2_	97	±	1.3 ^a^_1_	29	±	5.0 ^b^_2_
70 ppm	97	±	1.3 ^a^_1_	0	±	0.0 ^b^_3_	64	±	3.6 ^a^_3_	8	±	1.0 ^b^_3_	78	±	6.5 ^a^_3_	0	±	0.0 ^b^_2_	97	±	0.3 ^a^_1_	0	±	0.1 ^b^_2_

* SD: standard deviation. Superscript (a, b): mean values with different letters indicate significant differences (*p* < 0.05) between extraction methods within the same sample. Subscript (1, 2, 3): mean values with different numbers indicate significant differences (*p* < 0.05) among extract concentrations within the same extraction method.

## Data Availability

The original contributions presented in this study are included in the article. Further inquiries can be directed to the corresponding author.
